# Quantifying phenylhydrazine-induced Heinz body formation in normal human erythrocytes by flow cytometry versus microscopy or spectrophotometry

**DOI:** 10.1093/biomethods/bpag047

**Published:** 2026-08-28

**Authors:** Mewahyu Dewi, Sri Widia Azraki Jusman, Alida Roswita Harahap, John Kevin Baird

**Affiliations:** Doctoral Program in Biomedical Science, Faculty of Medicine, Universitas Indonesia, Jakarta 10430, Indonesia; Oxford University Clinical Research Unit Indonesia, Jakarta 10430, Indonesia; Doctoral Program in Biomedical Science, Faculty of Medicine, Universitas Indonesia, Jakarta 10430, Indonesia; Department of Biochemistry and Molecular Biology, Universitas Indonesia, Jakarta 10430, Indonesia; Doctoral Program in Biomedical Science, Faculty of Medicine, Universitas Indonesia, Jakarta 10430, Indonesia; Oxford University Clinical Research Unit Indonesia, Jakarta 10430, Indonesia; Nuffield Department of Medicine, Centre for Tropical Medicine & Global Health, University of Oxford, Oxford, OX3 7LF, United Kingdom

**Keywords:** G6PD deficiency, haemolytic toxicity, phenylhydrazine, Heinz bodies

## Abstract

Heinz bodies occur in patients experiencing specific drug-induced acute haemolytic crises, most often in those with an inherited deficiency in glucose-6-phosphate dehydrogenase activity. Laboratory methods for quantifying Heinz bodies in *in vitro* systems include direct microscopic counts from crystal violet-stained thin blood films or spectrophotometric measurement of free crystal violet in RBC suspensions treated for Heinz body induction. Each method is relatively laborious and prone to errors. In this study, we sought to evaluate and validate an improved flow cytometry-based method for detecting Heinz bodies in human red blood cells after *in vitro* exposure to phenylhydrazine using washed RBCs. We report good concordance between flow cytometric counts of Heinz bodies and microscopic and spectrophotometric analyses of the same suspensions. Flow cytometry offers a less laborious, more precise, and more accurate quantification of Heinz body formation, with strong potential to improve the ease of use in experimental applications.

## Introduction

Over 400 million people are affected by glucose-6-phosphate dehydrogenase (G6PD) deficiency [1]. Most such people are at risk of *Plasmodium vivax* malaria and require therapy involving 8-aminoquinolines (8-AQ) such as primaquine or tafenoquine. These drugs are the only therapeutic option against latent vivax malaria, and they provoke serious acute haemolytic anaemia in G6PD-deficient patients [2, 3]. Aggregates of degraded haemoglobin called Heinz bodies are 2–3 µm micellar clusters found in erythrocytes of patients who are G6PD-deficient and in haemolytic crisis, with their presence first linked to 8-aminoquinoline toxicity in the 1920s [4]. These inevitably occur in patients undergoing an 8-AQ-induced haemolytic crisis [5] and have been thoroughly examined in animals and *in vitro* systems using phenylhydrazine (PH) or some of its analogues [6, 7].

The mechanism of the haemolytic toxicity of 8-AQ remains unclear, with the suggested explanation involving the metabolic conversion of these drugs to 5-hydroxylated species by cytochrome P450 isozyme 2D6 (CYP2D6), which then produces quinonimine derivatives and ultimately leads to the formation of Heinz bodies and acute haemolytic anaemia [8, 9]. In rats, PH-induced haemolytic anaemia occurs [6], in which the phenyl group of the toxin covalently attaches to the porphyrin ring of haemoglobin, leading to the formation of irreversible ferrihaemochrome. Accurate and cost-effective measurement of Heinz bodies would greatly assist laboratory explorations of 8-AQ haemolytic toxicity. Such a robust and validated platform would allow ranking of these substances by relative haemolytic toxicity during preclinical testing. That same exploration may also shed light on the mechanism of 8-aminoquinoline haemolytic toxicity in G6PD-deficient humans.

In the current study, we evaluated and slightly modified the methodology described by Palasuwan *et al.* [10] for flow cytometric quantification of Heinz bodies in PH-treated G6PD-normal human red blood cell suspensions, and compared it with microscopic and spectrophotometric methods. This work aimed to optimize and validate the quantification of Heinz body formation induced by exposing human red blood cells to PH *in vitro*.

## Materials and methods

### Ethical approval

The study participants were recruited according to the ethical protocol 23-03-0384 approved by OxTREC Reference: 567-23, and the Health Research Ethics Committee—Faculty of Medicine Universitas Indonesia and Dr. Cipto Mangunkusumo National Hospital (HREC FMUI-CMH) (Komite Etik Penelitian Kesehatan Fakultas Kedokteran Universitas Indonesia—RSUP Nasional Dr Cipto Mangunkusumo, KEPK FKUI-RSCM) with Ethic Approval Letter KET-644/UN2.F1/ETIK/PPM.00.02/2023, dated 22 May 2023. The participants were informed about the study, and informed consent was obtained before they donated their blood.

Samples were obtained from three healthy adult male volunteers with normal G6PD activity. Each volunteer had his blood drawn three times in 3 consecutive weeks. Blood was treated and measured fresh on the same day.

### Heinz body induction with phenylhydrazine

Venous whole blood samples were collected into tubes containing K2EDTA (Golden Vac, K2EDTA, 3 ml, GD030EK2). Red blood cells (erythrocytes) were separated by centrifugation (755 × g, 10 min, 4°C). The buffy coat and plasma were removed, and the sample was washed 2× with 0.1 mM PBS (pH 7.4). Erythrocyte number was measured using an automatic haematology analyser (DYMIND-DF50 Auto Haematology Analyser).

Phenylhydrazine hydrochloride (PH) (Aldrich, 114715-5G) was prepared fresh in every experiment. PH working solution was prepared at a concentration of 50 mM in 0.1 mM phosphate-buffered saline (PBS) (pH 7.4). The erythrocyte suspension and PH were then prepared to yield final treatment concentrations ranging from 0 (PBS without PH added) to 1.0 mM. All three participants with normal G6PD levels were washed and treated with PH, with each reaction containing 10^6^ cell/µl erythrocytes per reaction to standardize the conditions.

The mixtures were incubated in a water bath at 37°C for 40 min. Afterwards, erythrocytes were washed once with PBS to remove PH and stop Heinz body formation. The erythrocyte suspensions were then prepared immediately for analysis, which included Heinz body measurement via microscopy, spectrophotometry, or flow cytometry, depending on the method used.

### Measurement of Heinz bodies using microscopy and spectrophotometry methods

The treated erythrocyte suspensions were stained with crystal violet (CV) (V5262-250 ml; Sigma) to facilitate visualization under a light microscope. Ten millilitre of 0.04% CV was added to 0.5 ml of the treated erythrocyte suspensions, and the mixture was incubated at 37°C for 1 h. The cell suspensions were then centrifuged at 3000 × g for 10 min. The supernatant was carefully discarded, and 3 µl of the pellet was spread onto a clean glass slide to form a thin blood film. Slides were air-dried and stored for examination. Each count was performed in triplicate, with 1000 erythrocytes examined per sample [10].

The spectroscopic methods are adapted from those described by Summerfield and Tudhope [11]. In brief, the method measures the CV remaining after the reaction with Heinz bodies. More abundant Heinz bodies remove more CV from the solution, that is, fewer dyes result in more Heinz bodies. Washed erythrocyte suspensions treated as detailed above are added to a 2.5× volume of ice-cold water, held on ice for 15 min, then incubated in a water bath at 37°C for 15 min. The process was repeated once, and if there was no visual trace of red colour (complete haemolysis), the sample was finally washed with PBS. CV was added to a final concentration of 0.025% and incubated at 37°C for 1 h. Tubes were then centrifuged (10 000 × g, 10 min), and the absorbance of the supernatant was measured at 580 nm using cuvettes with a 10-mm light path on an Implen NanoPhotometer. The difference in absorbance (Δ*A*580 nm) between the control lacking Heinz bodies and the test mixtures provides a quantitative estimate of the relative quantity of Heinz bodies in the mixture. All samples were measured in duplicate.

### Flow cytometry quantification of the Heinz bodies

The flow cytometer methods are adapted from Palasuwan *et al.* [10]. Aliquots of 5 µl of PH-treated erythrocyte suspensions (as above) are added to 1000 µl PBS. The instrument employed is BD FACS Canto II (RRID: SCR_018056). During the initial optimization phase, a single healthy blood sample was subjected to varying concentrations of PH and analysed in duplicate to establish assay parameters. The assay evaluated the entire RBC population regardless of RBC size, as RBC dimensions did not differ significantly with the presence of Heinz bodies. Analysis focused primarily on signal intensity versus count.

Appropriate gating strategies were employed to distinguish the untreated RBC populations from those treated with 1 mM PH. The blue colour channel was used, with the best detection using PerCP-Cy5.5A (488 nm). To validate the gate setting, we examined three erythrocyte suspensions as controls in the preliminary study: (i) those lacking Heinz bodies; (ii) those with 100% Heinz body-positive erythrocytes; (iii) a 1:1 mixture of Heinz body-negative and -positive erythrocytes. Histograms from these experiments confirmed appropriate separation and signalling between Heinz body-negative and Heinz body-positive erythrocytes. Gating is thus used to determine the proportion of Heinz body-containing erythrocytes in the samples. All samples were measured in duplicate.

## Results and discussions

Normal G6PD activity subjects have given their consent to donate their blood and have enrolled in this study. The G6PD activity was confirmed to be normal, and no other blood disorders were detected by the osmotic fragility test. The haematology profiles of the subjects are summarized in Table 1.

**Table 1:** Haematological profile and G6PD activity of donors.

Haematology parameters	G6PD normal, mean ± SD
Hemoglobin (g/dl)	15.46 ± 2.364
Hematocrit (%)	42.88 ± 6.310
MCV (fl)	79.56 ± 7.708
MCH (pg)	28.68 ± 2.787
Erythrocyte count (×10^6^/ml)	4.936 ± 1.051
WBC (/ml)	8368 ± 1031
G6PD activity (U/gHb)	6.9 ± 0.755

All three participants with normal G6PD blood were washed and treated with PH, each containing 10^6^/µl erythrocytes per reaction. The proportion of Heinz bodies in samples was measured using three different methods and instruments: microscopy, spectrophotometry, and flow cytometry.

### Microscopy method

**Figure 1:**
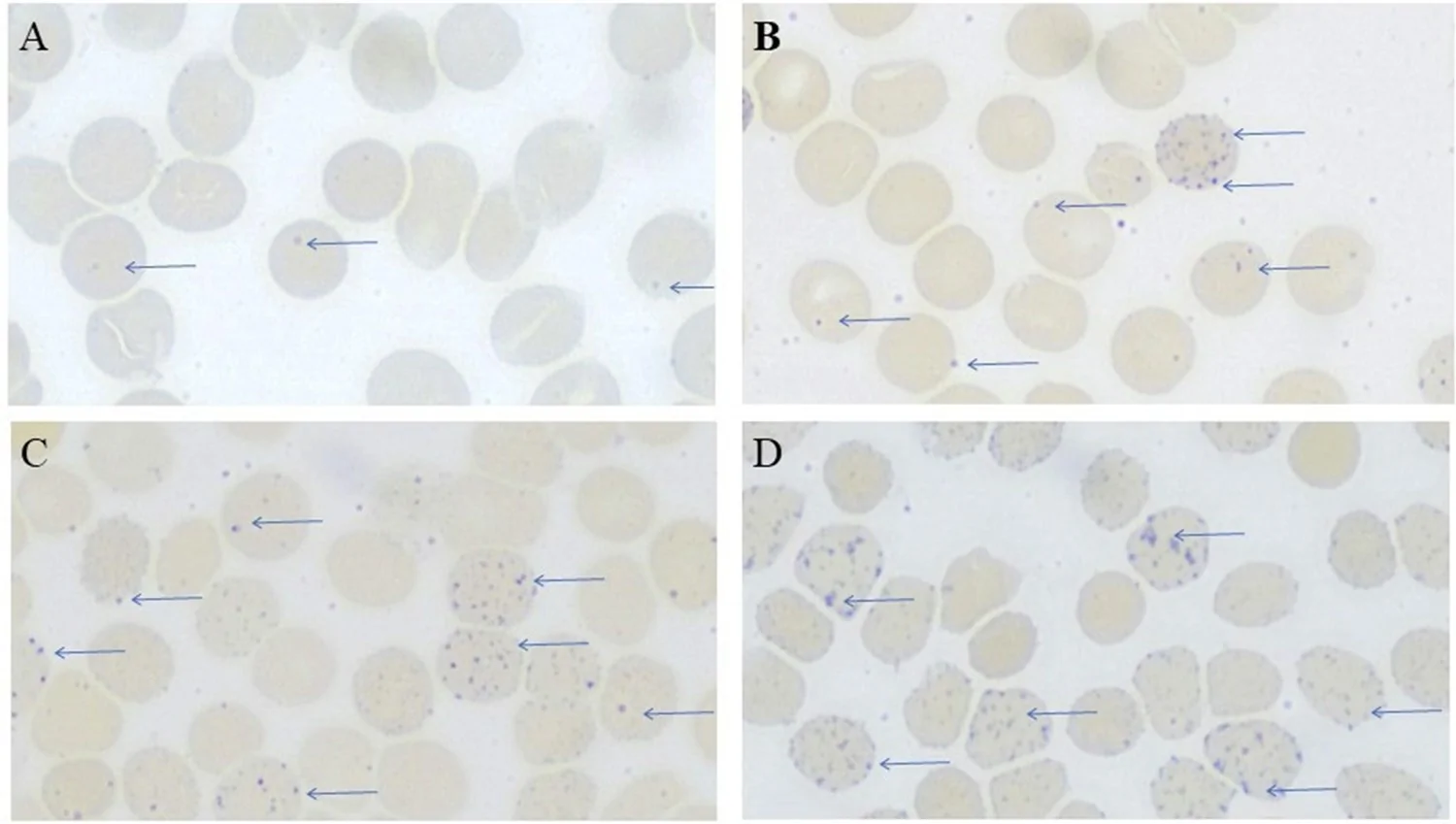
Blood film showing Heinz bodies at various PH concentrations (A) 0, (B) 0.2, (C) 0.5, and (D) 1.0 mM.

Heinz bodies appear as blue dots on a blood slide stained with CV. The number of Heinz bodies may vary from 1 to 2 to many Heinz bodies in one erythrocyte (Fig. 1). The Heinz body can only be seen in 1000× magnification (high-power field) with oil immersion.

The proportion of cells containing Heinz bodies per 1000 erythrocytes was counted following treatment with different concentrations of PH and is presented in Fig. 5.

### Spectrophotometry method

**Figure 2:**
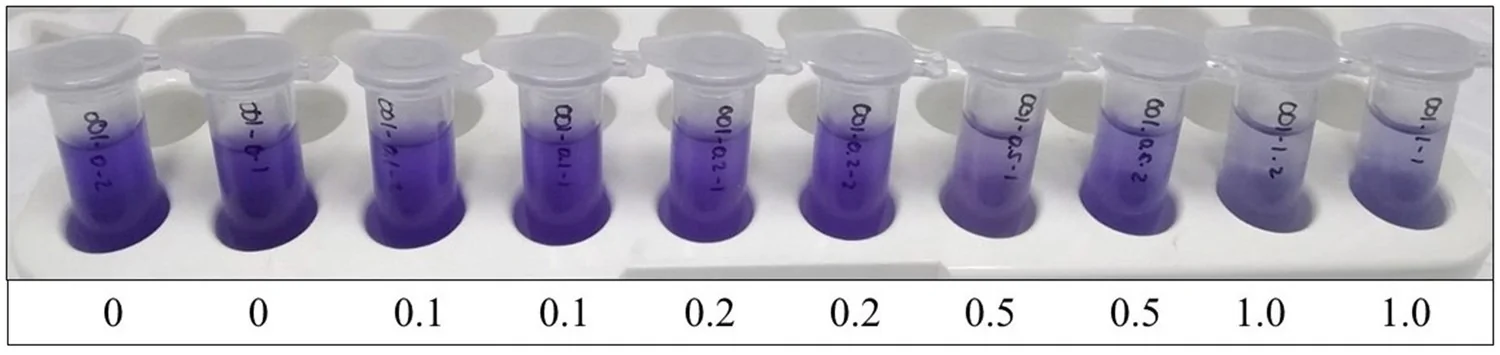
Supernatant with different violet colour intensities measured by spectrophotometer at 580 nm with PH concentrations of 0, 0.1, 0.2, 0.5, and 1.0 mM in duplicate. The gradation ranges from the darkest (high intensity = few Heinz bodies) to the lightest (lowest intensity = abundant Heinz bodies).

Heinz bodies absorb CV, removing it from the solution. Therefore, an abundance of Heinz bodies in a sample will yield a lower solution intensity than fewer Heinz bodies when measured at 580 nm in the spectrophotometer [11]. Figure 2 shows the visual difference in CV colour intensity as a function of pH concentration, which qualitatively reflects the number of Heinz bodies in the solution. Lower PH concentrations produced correspondingly higher levels of CV absorbance.

The spectrophotometric method provides a quantitative measurement of CV absorbance, which serves as a proxy for Heinz body formation. To approximate Heinz body formation from the spectrophotometric measurements, absorbance values were converted to the proportion of Heinz bodies in the sample. Preliminary experiments showed that almost all erythrocytes treated with 1 mM PH developed Heinz bodies. Therefore, the median absorbance at 1 mM PH in this study was considered an approximation of complete Heinz body formation (i.e. 100% of erythrocytes contain Heinz bodies). Thus, the proportion of Heinz bodies in the spectrophotometric method is counted as follows:


$$\%\mathrm{Heinz}\;\mathrm{body}=\;\frac{A_{580}\;{\mathrm{CV}}_{\mathrm{blank}}-A_{580}\;{\mathrm{CV}}_{\mathrm{PH}\;\mathrm{treated}}}{\mathrm{median}\;A_{580}\;{\mathrm{CV}}_{\mathrm{PH}\;1mM}}\times 100\%$$

*A*_580_ CV is the absorbance of the CV in the supernatant, measured at the wavelength of 580 nm. Blank is the CV dissolved in the buffer with the same concentration as the treatment; PH-treated is the sample treated with different concentrations of PH, and PH 1 mM is the sample treated with 1 mM of PH to induce 100% of Heinz bodies in the sample.

The result is plotted in Fig. 5, which illustrates Heinz body formation in erythrocyte suspensions at increasing PH concentrations, as measured by the optimized and validated spectrophotometric methodology.

#### Flow cytometry method

**Figure 3:**
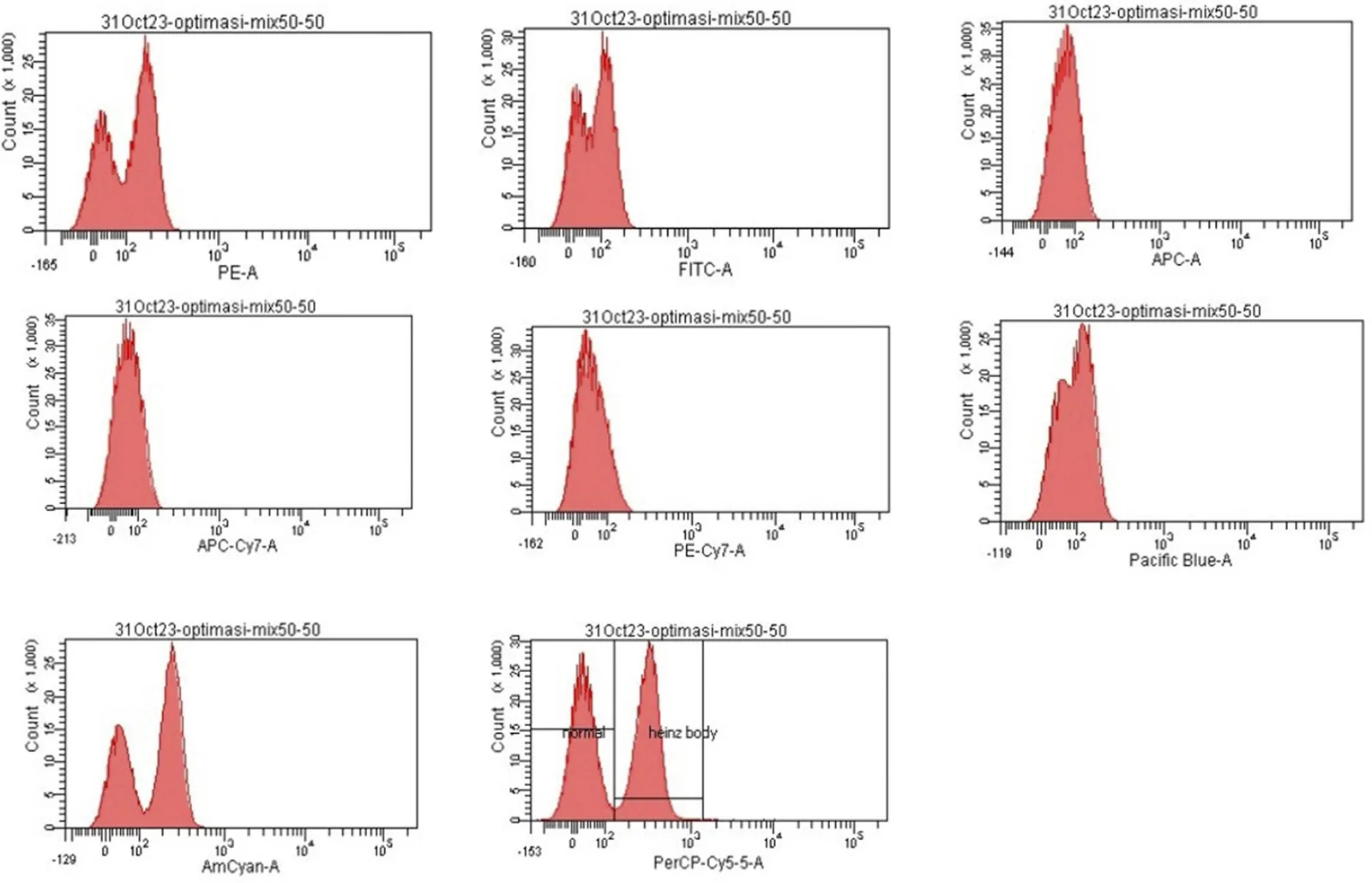
Autofluorescence from Heinz bodies, captured by different colour channels on the FACS Canto II, yielded different results for differentiating erythrocytes containing Heinz bodies in the samples.

The flow cytometry method was optimized using the BD FACS Canto II, which offers multiple colour channels. Channel selection was based on the ability to distinguish and quantify a 1:1 mixture of untreated erythrocytes (no Heinz bodies) and those treated with 1 mM PH (100% Heinz body-containing erythrocytes). Both AmCyan-A and PerCP-Cy5.5-A channels could separate the two populations, but only PerCP-Cy5.5-A accurately estimated cell population composition. Therefore, the PerCP-Cy5.5-A channel and its gating were used in this study (Fig. 3).

**Figure 4:**
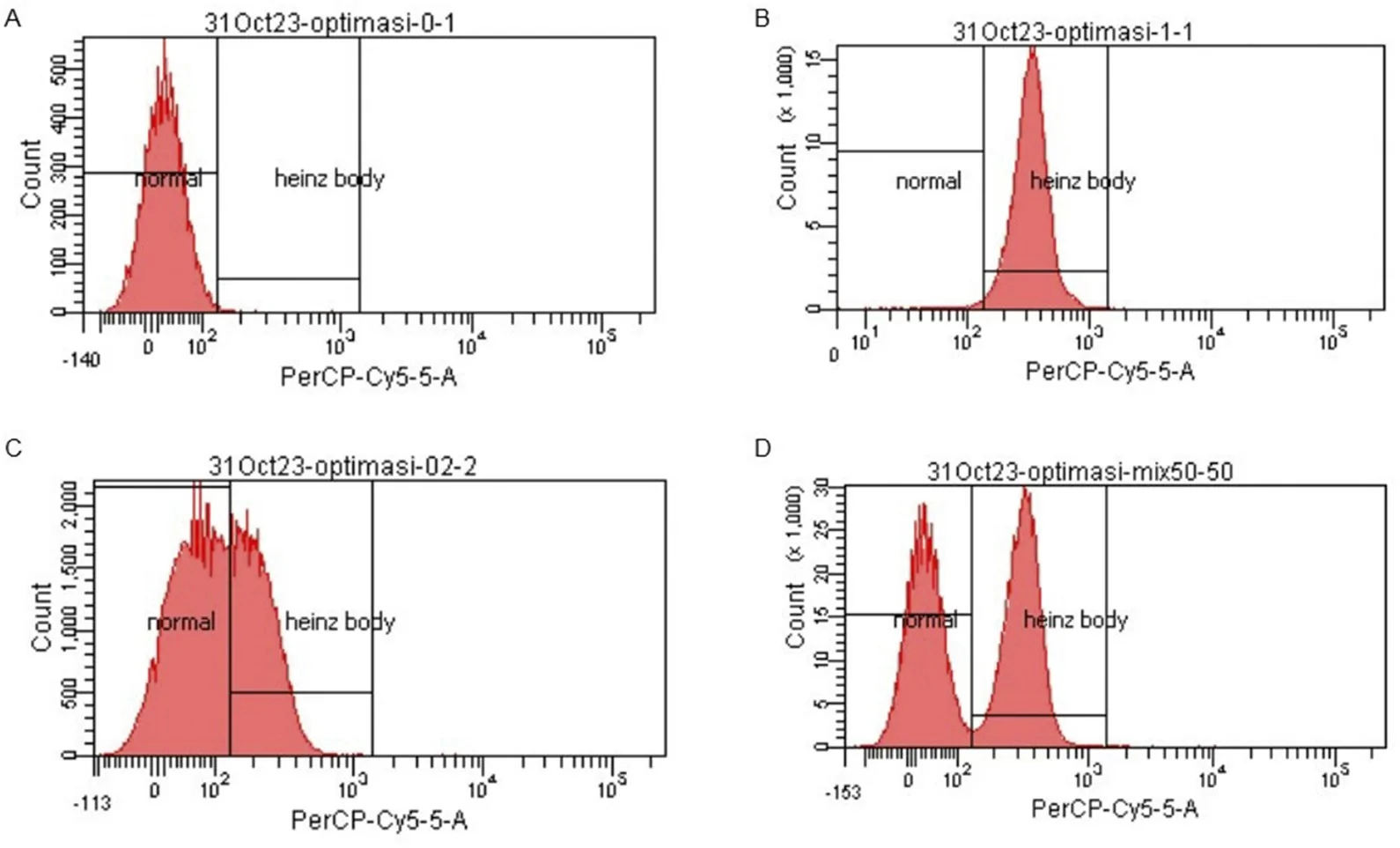
Optimized gating with the perCP-Cy5.5-a colour channel. The histogram of treated erythrocytes is as follows: A. 0 mM PH, B. 1 mM PH, C. 0.5 mM PH, D. Mix 1:1 of 0 mM and 1 mM PH. Abbreviation: PH, phenylhydrazine.

Gating was established using measurements from samples treated with varying concentrations of PH. The histogram demonstrates distinct peaks for the non-Heinz body (0 mM PH) and all-Heinz body (1 mM PH) erythrocyte samples (Fig. 4A and B). At lower PH concentrations (0.2 mM), the peak shifted rather than splitting into two populations (Fig. 4C). Accurate gating is therefore important for the precise quantification of erythrocytes containing Heinz bodies. Optimized gating on a 1:1 mixture of untreated and 1 mM PH-treated erythrocytes was used to distinguish normal from Heinz body-containing cells (Fig. 4D).

**Figure 5:**
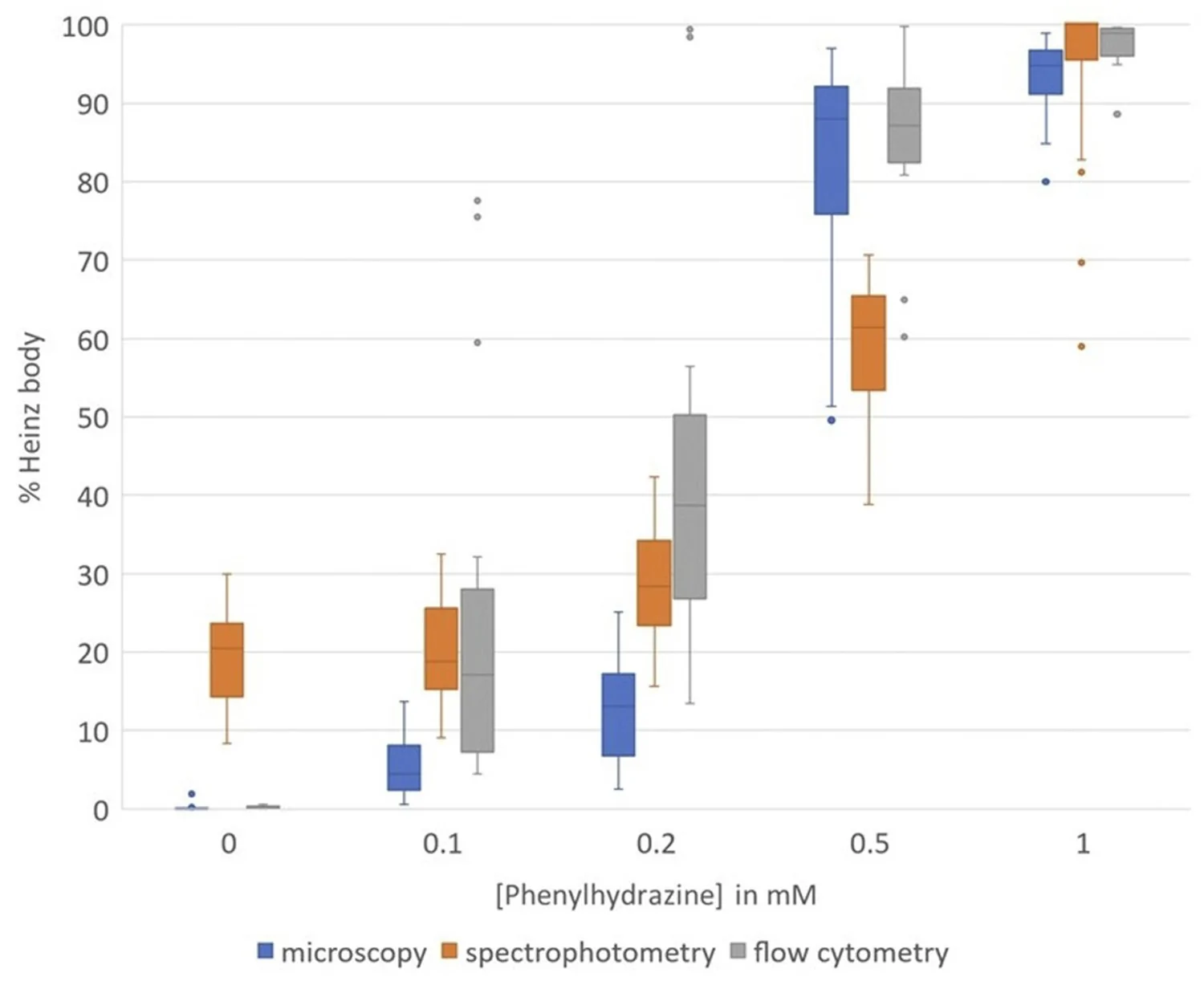
A plot of individual estimations of Heinz body formation in the blood from three volunteers measured simultaneously over 3 consecutive days of experimentation. The bars with limits are 95% CIs; the boxes are the first quartile, median, and third quartile. The dots outside the box are statistical outliers.

Using the optimized gating, the Heinz body proportion in samples treated with different PH concentrations is plotted in Fig. 5.

### Comparison of Heinz body measurement

The comparison of Heinz body measurements using three different methods is presented in Table 2 and Fig. 5. Across all methods, the proportion of Heinz bodies increased in a concentration-dependent manner with PH. However, inter-method agreement was highest at the upper PH concentration. The relative increases in Heinz body formation estimates at the applied PH concentrations were essentially similar among the three methods.

**Table 2:** Heinz bodies proportion based on the mean of three different methods.

[PH] in mM	Microscopy (%)	Spectrophotometry (%)	Flow cytometry (%)
0.0	0.44	20.99	0.26
0.1	3.01	22.10	27.57
0.2	34.32	29.76	43.30
0.5	88.84	64.28	85.59
1.0	97.07	99.24	97.60

The microscopy method (adapted from Palasuwan *et al.*) [10] is the most commonly used method for estimating Heinz body formation. However, this method proved relatively time-consuming; it took more than 1 h to stain and prepare the slide, and another 30 min to properly read each of the many slides. It required a well-trained eye to distinguish Heinz bodies from reticulocytes (containing stained reticulum) in the blood samples. When the data did not agree, another microscopist was employed to provide a third count. Although laborious and time-consuming, the microscopy method provides a direct quantification of Heinz bodies and does not require the more sophisticated equipment used in the other two methods.

The spectrophotometric method developed by Summerfield and Tudhope [11] provides quantitative absorbance values that serve as proxies for relative Heinz body formation. This approach estimates the total Heinz body content in the sample, regardless of whether the inclusions remain within erythrocytes or have been released into the solution following erythrocyte lysis. The other downside of this method is that it measures all Heinz bodies that remain attached to the erythrocyte membrane, which may contain multiple Heinz bodies, especially at higher PH concentrations. Therefore, this method does not measure the frequency of erythrocytes containing Heinz bodies; it only estimates total Heinz body formation across the entire sample.

The flow cytometric method developed by Palasuwan *et al.* [10] may be the least laborious and most direct of the three methods analysed here. The sample can be measured directly in the flow cytometer after treatment, in the pre-gating channel, using the PerCyp5.5-A stored method. Data analysis indicates that flow cytometry provided better precision, especially at lower PH concentrations (0.1 and 0.2 mM), and yielded results similar to the microscopy method at 0.5 and 1 mM (Fig. 5).

The results from the three methods are compared statistically using STATA and analysed using the intraclass correlation coefficient (ICC) (Table 3). This method is used to assess how closely units within the same group resemble each other, or how consistent repeated measurements are across different instruments.

**Table 3:** ICC among the methods with the same concentrations of PH.

[PH] in mM	ICC	95% CI	Interpretation
Comparison among all concentrations	0.723	0.68–0.76	Good agreement
0.0	0.452	0.31–0.58	Poor-fair
0.1	0.498	0.35–0.62	Poor-fair
0.2	0.612	0.48–0.72	Moderate
0.5	0.851	0.79–0.90	Excellent
1.0	0.894	0.84–0.93	Excellent

Overall, the three methods show good agreement (ICC = 0.723), and the proportion of Heinz bodies increases with PH concentration. At the lowest concentration (0.1 mM PH), the ICC is low (<0.5), and the correlation among methods is considered fair to poor. As the concentration increases, the agreement rises, as shown at PH concentrations of 0.5 and 1 mM, with excellent agreement.

A Bland-Altman (bias) analysis is then performed to further analyse the agreement between each pair of measurement methods (Table 4). This method measures the bias (mean difference) and the limits of agreement (LoA). LoA is defined as the range between the lower and upper limits within which most differences between two measurements fall. The lower LoA and bias indicate greater agreement between the two methods.

**Table 4:** Bland-Altman analysis between each pair of measurement methods.

Pair of measurement	Bias (mean diff)	SD Diff	95% lower LoA	95% upper LoA	LoA	*P* value
Microscopy versus spectrophotometry	−0.092	0.312	−0.680	0.550	1.230	<.001
Microscopy versus flow cytometry	−0.125	0.335	−0.780	0.530	1.310	<.001
Spectrophotometry versus flow cytometry	−0.060	0.287	−0.620	0.500	1.120	<.001

LoA is the range between the lower and upper limits within which most of the differences between two measurements fall.

The three methods show significant differences (*P* values <.001). Microscopy, as the gold standard, shows better agreement with spectrophotometry (LoA = 1.230) than with flow cytometry (LoA = 1.310). However, among all pairs, spectrophotometry and flow cytometry have the highest agreement, with the lowest bias (−0.06) and LoA (1.120). The conclusion from this analysis is that all pairs exhibit a wide range of LoA, indicating that the three methods are not directly interchangeable, especially at low PH concentrations.

Both spectrophotometry and flow cytometry detected higher levels of Heinz bodies at low PH concentrations (treated with 0.1 and 0.2 mM) than microscopy. However, the spectrophotometric method lacks accuracy because it detects all Heinz bodies, whether within intact cells or released from lysed cells. The proportion of cells bearing Heinz bodies cannot be determined by spectroscopy.

## Conclusion

Based on our findings, all methods resulted in the same proportion of Heinz bodies when treated with a higher concentration of PH. Flow cytometry is more sensitive than other methods for detecting a low proportion of Heinz bodies in intact erythrocytes. It offers an advantage by producing results in much less time, although it requires more sophisticated technology and skills. Spectrophotometry can qualitatively compare Heinz bodies across samples. It is a good measure of the total Heinz body formation, whether intact or dispersed in solution. It may be useful in estimating the potential for haemolytic toxicity, but it requires a standard (microscopy method) to quantify and estimate the extent of Heinz body formation in relation to optical absorbance in any given sample. The method of choice among these approaches will hinge on needs and capabilities.

## Data Availability

Data are not publicly available.
